# Pharmacologic inhibition of NLRP3 reduces the levels of α-synuclein and protects dopaminergic neurons in a model of Parkinson’s disease

**DOI:** 10.1186/s12974-023-02830-w

**Published:** 2023-06-22

**Authors:** Jesus Amo-Aparicio, Jonathan Daly, Jesper Falkesgaard Højen, Charles A. Dinarello

**Affiliations:** 1 Department of Medicine, University of Colorado, 12700 E 19th Ave, Aurora, CO 80045 USA; 2 Department of Clinical Medicine, Aarhus University, 8200 Aarhus, Denmark

**Keywords:** Inflammasome, NLRP3, α-synuclein, OLT1177, Parkinson’s disease

## Abstract

**Background:**

Parkinson’s disease (PD) is characterized by a progressive degeneration of dopaminergic neurons, which leads to irreversible loss of peripheral motor functions. Death of dopaminergic neurons induces an inflammatory response in microglial cells, which further exacerbates neuronal loss. Reducing inflammation is expected to ameliorate neuronal loss and arrest motor dysfunctions. Because of the contribution of the NLRP3 inflammasome to the inflammatory response in PD, we targeted NLRP3 using the specific inhibitor OLT1177^®^.

**Methods:**

We evaluated the effectiveness of OLT1177^®^ in reducing the inflammatory response in an MPTP neurotoxic model of PD. Using a combination of in vitro and in vivo studies, we analyzed the effects of NLRP3 inhibition on pro-inflammatory markers in the brain, α-synuclein aggregation, and dopaminergic neuron survival. We also determined the effects of OLT1177^®^ on locomotor deficits associated with MPTP and brain penetrance.

**Results:**

Treatment with OLT1177^®^ prevented the loss of motor function, reduced the levels of α-synuclein, modulated pro-inflammatory markers in the nigrostriatal areas of the brain, and protected dopaminergic neurons from degeneration in the MPTP model of PD. We also demonstrated that OLT1177^®^ crosses the blood–brain barrier and reaches therapeutic concentrations in the brain.

**Conclusions:**

These data suggest that targeting the NLRP3 inflammasome by OLT1177^®^ may be a safe and novel therapeutic approach to arrest neuroinflammation and protect against neurological deficits of Parkinson’s disease in humans.

**Supplementary Information:**

The online version contains supplementary material available at 10.1186/s12974-023-02830-w.

## Introduction

Parkinson’s disease (PD) is a neurodegenerative disorder characterized by a progressive impairment of voluntary motor control producing tremors, rigidity, and bradykinesia [1]. PD is the second-most frequent neurodegenerative disease after Alzheimer’s disease (AD). Currently, the prevalence of PD is 2–3% in world populations over 65 years of age, being twice as common in men as in women in most populations [2]. PD is mostly sporadic with less than 10% of the cases being inherited [3]. From 1990 to 2015, the number of subjects with PD doubled to 6 million worldwide [4]. This number is projected to double to over 12 million by 2040 as average life expectancy increases [5, 6].

The pathological hallmark of PD is the loss of dopaminergic neurons in the substantia nigra pars compacta (SNpc) resulting in reduced levels of the neurotransmitter dopamine (DA) in the striatum. Since DA plays a role in the modulation of some brain circuits related to movement, its reduction leads to motor impairments [7]. Although most therapeutics focus on the re-establishment of DA levels in the brain, the damage persists and expands, and treatments are unable to effectively slow or halt disease progression [5]. Therefore, there is a need to develop pharmacological strategies for preventing disease progression.

The aggregation of the intracellular α-synuclein is considered the primary pathogenic mechanism of dopaminergic neuron death [8, 9]. In the healthy brain, constitutive α-synuclein appears at the pre-synaptic terminals of the neurons and participates in the regulation of neurotransmitter release, synaptic function, and plasticity [10]. However, misfolded fibrillar α-synuclein, in the form of Lewy bodies, is found inside dead or dying neurons [10]. The specific molecular mechanisms for the aggregation and neurotoxicity of α-synuclein remain unknown, although further advances have been made [11].

In addition to the role of α-synuclein in the pathogenies of PD, neuroinflammation also contributes to the degeneration of dopaminergic neurons [12]. Increased levels of pro-inflammatory cytokines are found on post-mortem nigrostriatal dopaminergic regions in patients with PD [13, 14]. Damage-associated molecular patterns (DAMPs) from dying dopaminergic neurons contribute to microglial activation in the SNpc of PD patients [15]. Activated microglia promote additional damage via the release of pro-inflammatory cytokines and neurotoxic factors [16]. Hence, modulation of inflammatory response is a promising approach for mitigating the symptoms of PD.

A paramount of the mechanisms of inflammation is the NOD-like receptor pyrin domain containing protein 3 (NLRP3) inflammasome. NLRP3 is an intracellular macromolecular complex involved in the first stages of the inflammatory response [17–19]. NLRP3 senses and is activated by a wide variety of stimuli including Toll-like receptors, RNA viruses, extracellular ATP, and cytokines [20]. NLRP3 activation results in oligomerization and the conversion of inactive caspase-1 to catalytically active caspase-1 [21]. Active caspase-1 initiates the downstream processing and release of pro-inflammatory cytokines IL-1β and IL-18 [21]. In PD, recent findings suggest that NLRP3 inflammasome activation is critical for dopaminergic neurodegeneration [22, 23]. Moreover, a lack of NLRP3 prevents motor dysfunction [22] and protects dopaminergic neurons in a mouse model of PD [23].

OLT1177 is a small synthetic compound (MW 133.17 g/mol) that selectively inhibits the oligomerization and activation of the NLRP3 inflammasome [24]. OLT1177 is safe for humans [25, 26] and has been shown to reduce disease severity in neurodegenerative mouse models of multiple sclerosis [27], spinal cord injury [28], and AD [29]. In this study, we assessed the therapeutic effects OLT1177 on mice subjected to a 1-methyl-4-phenyl-1,2,3,6-tetrahydropyridine (MPTP) model of PD. We provide evidence that the pharmacologic inhibition of NLRP3 by OLT1177 mitigates the inflammatory response and reduces the levels of α-synuclein in the brain, resulting in the protection of dopaminergic neurons and improvement of locomotor performance.

## Materials and methods

### Ethics declarations

Animal protocols were approved by the University of Colorado Animal Care and Use Committee. Male wild-type C57BL/6J mice (9 weeks of age) were purchased from the Jackson Laboratory (Bar Harbor, ME, USA) and were housed in the animal facility for at least 7 days before use.

### MPTP administration

Two dosing regimens of MPTP (Sigma-Aldrich, St. Louis, MO, USA) were used: acute and subacute. For the acute regimen, MPTP was administered intraperitoneally (i.p.) four times in one day. Administrations were performed every 2 h over a 6-h period. For the subacute regimen, mice received a single i.p. administration of MPTP per day for five consecutive days. 20 mg/kg free-base MPTP was used for every injection in both models based on previous protocols [30]. Sterile saline was used as a vehicle. Control mice were injected with saline solution using the same administration protocol and defined as sham. Mice were killed at different days after the first MPTP injection.

### OLT1177 administration

OLT1177^®^ (dapansutrile, Olatec Therapeutics LLC, New York, NY, USA) was administrated i.p. one hour before the first MPTP injection. Two different doses of OLT1177 were used: 60 mg/kg and 200 mg/kg. OLT1177 was administrated once a day until the end of the experiment. Fresh solutions were prepared each day. Saline was used as vehicle.

### Locomotor evaluation

The rotarod test (Ugo Basile, Gemonio, Italy) was used to evaluate locomotor function in mice subjected to MPTP-acute administration. We used a rotarod device with a 3-cm cylinder rod and an accelerating program [31, 32]. Mice underwent a training period of three consecutive days prior to MPTP administration. In each training session, mice were subjected to four constant speeds (10 rpm, 20 rpm, 30 rpm, and 40 rpm) for 150 s. On the testing day, 3 days after MPTP, mice were placed on the rotarod device for 150 s at 30 rpm. Immediately after 150 s, the accelerating session started, and the speed increased from 30 to 70 rpm over a 500-s interval. The time to fall was recorded. Accelerating sessions were repeated 3 times with a resting interval of 5 min between sessions. The average time to fall per mouse was calculated and used for final measurement. 9 mice were used for the Sham + Saline group. 12 mice were used for the MPTP + Saline group. In the MPTP + OLT1177 group, 9 and 14 mice were used for the 60 mg/kg and 200 mg/kg doses, respectively.

### Culture of neonatal microglia

Primary microglial culture was prepared following previous protocols [33]. Briefly, brains of P3 pups were isolated and disaggregated to prepare a mixed glial culture. After a minimum of 10 days in culture, microglial cells growing on the top layer were collected and plated in a 96-well plate at a density of 50,000 cells/well. For knockdown of ATG5, silencing interfering RNAs (siRNA, Silencer siRNA, Invitrogen, Waltham, MA, USA) against the *Atg5* gene were transfected into microglial cells using Lipofectamine RNAiMAX transfection reagent (Invitrogen) following the manufacturer’s protocol. Experiments were performed 72 h post-transfection.

### Recombinant α-synuclein stimulation in vitro

Recombinant α-synuclein (rPeptide, Watkinsville, GA) was prepared according to previous protocols [34]. The lyophilized form was rehydrated to a concentration of 1 mg/ml and incubated for 3 days at 37 °C under constant agitation to induce aggregation. The aggregates were used to stimulate microglial cell in vitro at a 10 µM concentration. After 6 h, supernatants were collected, and cells were washed twice and lysed with RIPA buffer (ThermoFisher, Waltham, MA, USA) supplemented with protease inhibitor (ThermoFisher). Culture samples were analyzed by western blotting. To evaluate the clearance of α-synuclein, cells were stimulated with 10 µM of α-synuclein for 1 h-pulse, rinsed twice with fresh medium, and incubated in fresh medium for 5 additional hours [35]. In all the cases, cells were treated with 10 µM OLT1177 1 h before α-synuclein stimulation. The experiments were repeated 3 times.

For western blotting of culture samples, lysates and supernatants were resolved in a Mini-PROTEAN TGX 4–20% gradient gel (Bio-Rad, Hercules, CA, USA) in 0.1% SDS running buffer (Bio-Rad). The gel was transferred to a 0.45 μm PVDF membrane (Millipore, Burlington, MA, USA) and blocked with 5% blocking buffer (Bio-Rad) in TBS 0.1% (v/v) Tween solution for 1 h at room temperature. Membranes were incubated overnight with primary antibody against mouse NLRP3 (1:2000, AdipoGen, San Diego, CA, USA), p62 (1:1000, Cell Signaling, Danvers, MA, USA), LC3B (1:1000, Cell Signaling), and α-synuclein (1:1000, Cell Signaling). Peroxidase-conjugated secondary antibodies and chemiluminescence were used to detect the protein concentration. Conjugated antibody against β-actin (1:1000, Santa Cruz Biotechnology, Dallas, TX, USA) was used for quantification.

### α-synuclein and TREM2 measurements in brain homogenates

For western blotting of α-synuclein, mice were killed 24 h after the last MPTP-subacute administration. The ventral midbrain (VM) was excised and homogenized in RIPA buffer (Thermo Fisher) supplemented with protease inhibitor (Thermo Fisher) using a TissueRuptor (Qiagen, Germantown, MD). Protein concentration was determined using a BCA Protein Assay Kit (Thermo Fisher) according to the manufacturer’s instructions. Samples were diluted to 2 µg/µl in the same extraction buffer. For TREM2, mice were killed at day 4 after MPTP-acute administration. The striatum (St) was excised, and proteins were extracted and quantified as previously described.

30 μg of protein per sample were resolved in a Mini-PROTEAN TGX 4–20% gradient gel and transferred to a 0.1 μm nitrocellulose membrane (GE Healthcare, Chicago, IL, USA). Membranes were incubated overnight with primary antibody for mouse a-synuclein (1:1000, Cell Signaling) or TREM2 (1:1000, Cell Signaling). 4–5 mice per group were used.

### Cytokine measurement in brain homogenates

Mice were killed on day 4 and 7 after MPTP-acute administration. Brains were collected and the St and VM areas of both hemispheres were excised and frozen in liquid nitrogen. Samples were homogenized in RIPA buffer (Thermo Fisher) supplemented with protease inhibitor (Thermo Fisher) using a TissueRuptor (Qiagen). Protein concentration was determined using a BCA Protein Assay Kit (Thermo Fisher) according to the manufacturer’s instructions. Samples were diluted to 2 µg/µl in the same extraction buffer.

Levels of IL-1β, IL-18, IL-6, and IL-17A in brain homogenates were measured by ELISA DuoSet kit (R&D Systems, Minneapolis, MN, USA) according to the manufacturer’s directions. Absorbance was quantified in a microplate reader (Bio-Tek, Santa Clara, CA, USA). Final values were normalized by the protein concentration of each sample. 4–5 mice per group were used.

### TH, Iba1, and GFAP immunostaining

Seven days after MPTP-acute administration, mice were perfused with 4% paraformaldehyde (Sigma-Aldrich) in PBS. Brains were removed and immersed in the same perfusion buffer overnight and then cryoprotected with 30% sucrose in 0.1 M PBS at 4 °C. After dehydration, samples were embedded in paraffin molds for sectioning. Coronal brain sections (4-µm thick) containing the St and SNpc were obtained and transferred to glass microscope slides.

To assess the viability of dopaminergic neurons, nigrostriatal integrity was performed by tyrosine hydroxylase (TH) immunostaining. After antigen retrieval, slides were incubated with primary anti-TH antibody (1:2000, Abcam, Cambridge, UK) for 30 min at room temperature. TH antibody binding was detected using an anti-rabbit HRP-conjugated secondary polymer, followed by chromogenic visualization with diaminobenzidine (DAB, Cell Signaling). Hematoxylin counterstain was used to visualize nuclei. Dopaminergic immunoreactivity in the St was calculated by measuring the integrated density by NIH ImageJ software. Stereological counting in the SNpc was performed by manual quantification of TH-positive neurons. Three sections per mice were used. To assess microgliosis and astrogliosis, ionized calcium-binding adaptor molecule 1 (Iba1, 1:15,000, Abcam) and glial fibrillary acidic protein (GFAP, 1:200, Abcam) antibodies were used following the same protocol as before. The Bond Polymer Refine Red Detection Kit (Leica, Wetzlar, Germany) was applied for red chromogen in Iba1 staining. Processing of the samples was performed by Inotiv Boulder (Boulder, CO). 6–7 mice per group were used.

### Concentration of OLT1177 in brain homogenates and peripheral blood analysis

On day 7 after MPTP-acute administration, peripheral blood and brains were collected. Peripheral blood was obtained by enucleation using 0.5 M EDTA tubes. White blood cells, lymphocytes, monocytes, and granulocytes were measured using a HemaTrue cell counter (Heska, Loveland, CO). Samples were centrifuged at 5,000 rpm for 10 min to isolate the plasma section. VM was isolated from the brain. OLT1177 content in the plasma and VM homogenates was measured by tandem mass spectrometry at Syneos Health (Princeton, NJ). 5 mice per group were assessed.

### Statistics

All the analyses were conducted by GraphPad Prism 10 software. Kolmogorov–Smirnov test was used to test normality. One-way ANOVA with Tukey’s post hoc correction was used for most of the analyses. Kaplan–Meier test was used to evaluate the differences in the speed pattern at the rotarod device. Two-way ANOVA with Tukey’s post hoc correction was used for number of cells in the peripheral blood. Results were expressed as mean ± SEM and were considered significant at *p* < 0.05.

## Results

### OLT1177 rescues the locomotor impairments associated to MPTP

**Fig. 1:**
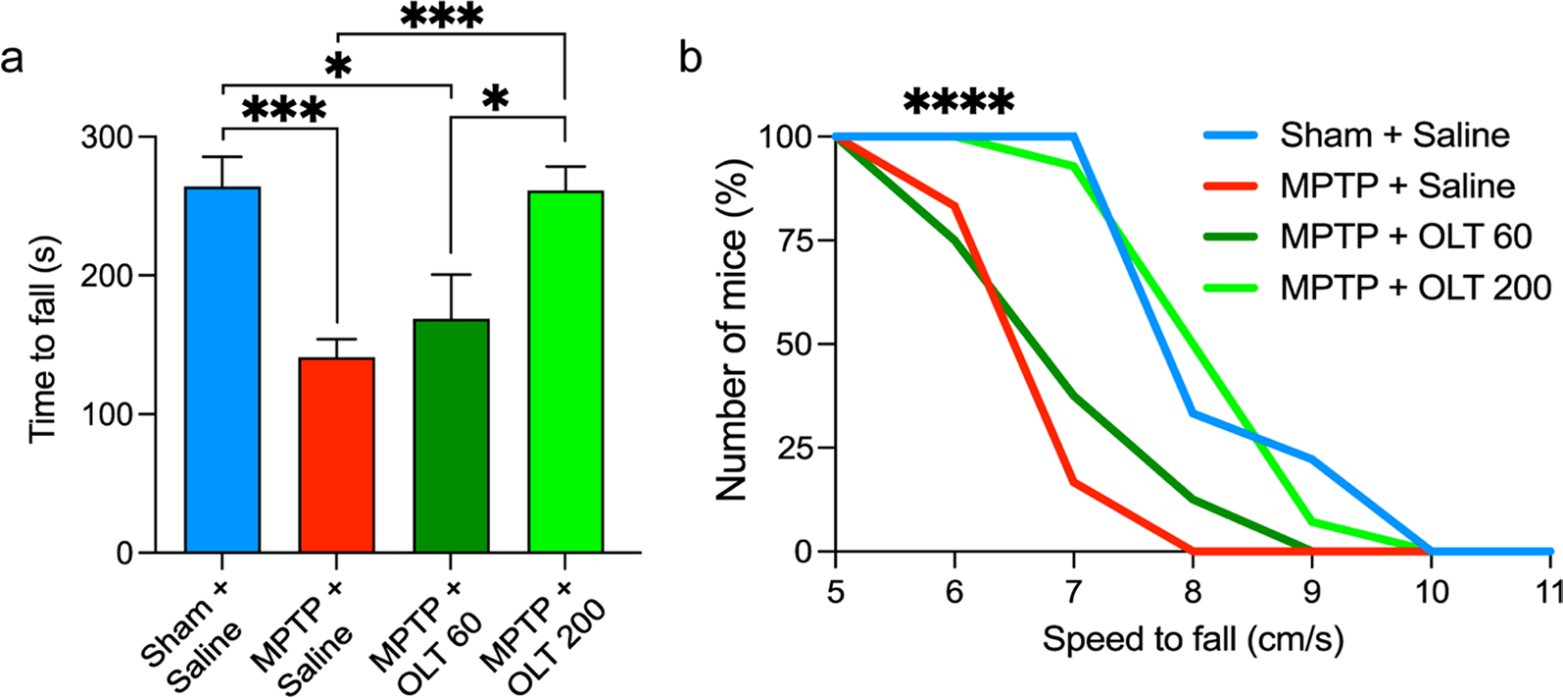
OLT1177 improves motor performance after MPTP-acute administration in mice. **a** Time to fall on the accelerating rotarod device 3 days after MPTP administration. Mice received OLT1177 either at 60 mg/kg (OLT 60) or 200 mg/kg (OLT 200) daily starting one hour before MPTP administration. Saline was used as vehicle. Sham mice that did not receive MPTP were used as control. **b** The running pattern of the mice at the different speeds on the accelerating rotarod device. Data are pooled from 3 separate experiments and are represented as the mean ± SEM for **a** and the mean for **b**. *N* = 9 for Sham + Saline, *N* = 12 for MPTP + Saline, *N* = 9 for MPTP + OLT 60, and *N* = 14 for MPTP + OLT 200. One-way ANOVA with Tukey’s post hoc correction was used to analyze differences between groups for **a** with *p < 0.05, **p < 0.01, and ***p < 0.001. Kaplan–Meier test was used to analyze differences between groups for **b** with ****p < 0.0001 for MPTP + OLT 200 against MPTP + Saline

First, we tested whether OLT1177 affected the motor performance of the mice after MPTP-acute administration. For this purpose, the locomotor skills of mice were assessed using the accelerating rotarod test [31, 32]. MPTP administration impaired the locomotor performance of the mice after three days, showing a significant reduction (120 s) in the time to fall in comparison with mice that did not receive MPTP (Fig. 1a). Approximately 50% of the mice in the MPTP + saline group failed on the rotating cylinder before the speed was increased to 7 cm/s; none reached 8 cm/s (Fig. 1b). Mice treated with a lower dose of OLT1177 (60 mg/kg) did not show any significant improvement in comparison with the MPTP + saline group, although a tendency to increase was observed (Fig. 1a, b). Treatment with a higher dose of OLT1177 (200 mg/kg) rescued locomotor deficits associated with MPTP by doubling (*p* < 0.001) the time to fall (Fig. 1a). Similar to the sham group, approximately 50% of the mice treated with the 200 mg/kg dose reached 8 cm/s and one of them reached 9 cm/s (Fig. 1b).

### OLT1177 crosses the blood–brain barrier and reaches therapeutic concentrations

Next, we tested the penetrance of OLT1177 into the brain. Following the same strategy as before, brain (VM) and plasma samples were collected on day 7 after MPTP-acute administration. Since we obtained better results with the highest dose of OLT1177 (200 mg/kg), we used this dose for the remainder of the experiments. 24 h after the last injection, OLT1177 reached 4 µg/g in the VM (Additional file 1: Fig. S1a). This is the same concentration observed in the plasma (Additional file 1: Fig. S1b), proving the crossing of the blood–brain barrier (BBB), and reaching concentrations sufficient to block the assembly of NLRP3 [24]. Hematological changes in peripheral blood were also evaluated. MPTP reduced the number of circulating white blood cells in the periphery, particularly lymphocytes. However, following the administration of OLT1177, the number of these cells significantly increased (Additional file 1: Fig. S1c).

### OLT1177 increases the clearance of α-synuclein in vitro

**Fig. 2:**
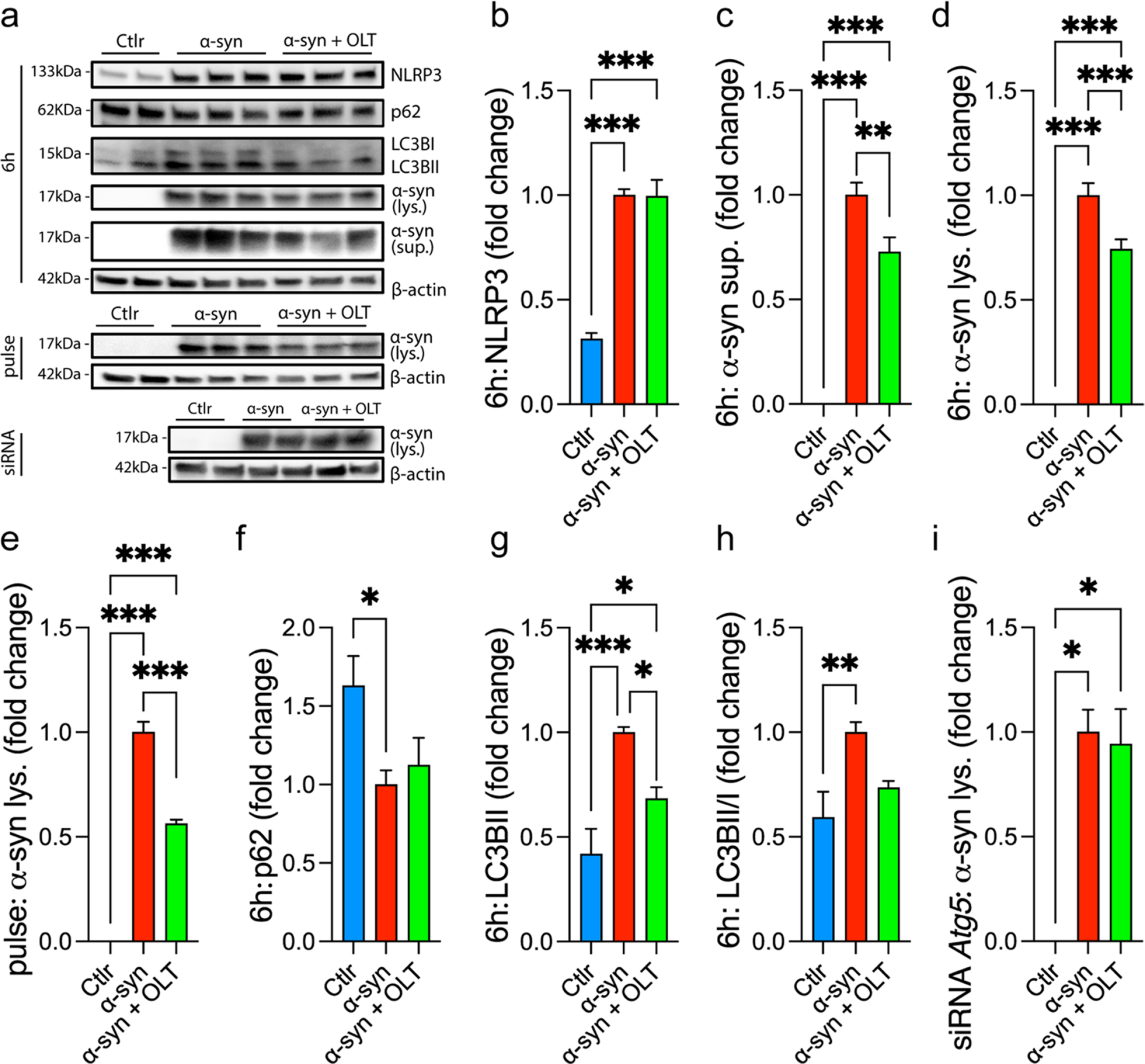
OLT1177 increases the clearance of α-synuclein by microglia in vitro. **a** Representative immunoblotting showing the levels of NLRP3, autophagy-related proteins, and α-synuclein in neonatal microglia. Cells were treated with OLT1177 (OLT, 10 µM) one hour before stimulation with recombinant α-synuclein (α-syn, 10 µM). β-actin was used as loading control. Molecular weight (kDa) is marked on the right side. **b**–**d** Quantification of the levels of NLRP3 (**b**), α-synuclein in the supernatant (**c**), and lysates (**d**) at 6 h. **e** Quantification of the levels of α-synuclein in the lysates of microglial cells after one-hour pulse of recombinant α-synuclein stimulation. **f**–**h** Quantification of the levels of p62 (**f)** and LC3B (**g**, **h)** at 6 h. **i** Quantification of the levels α-synuclein in the lysates after the blockade of *Atg5* gene by siRNA (**f**). Data are pooled from 3 separate experiments and are represented as the mean ± SEM. One-way ANOVA with Tukey’s post hoc correction was used to analyze differences between groups with *p < 0.05, **p < 0.01, and ***p < 0.001

To study the effects of OLT1177 on α-synuclein, neonatal microglia were cultured and stimulated with recombinant α-synuclein. As expected, α-synuclein increased the expression of NLRP3 in cell lysates (Fig. 2a, b). At 6 h post-stimulation, the levels of monomeric α-synuclein in both the supernatant (Fig. 2c) and the lysates (Fig. 2d) were reduced in cultures exposed to OLT1177 (10 µM, 1 h before), indicating an increased uptake and degradation of α-synuclein, respectively. Uptake of oligomers in the supernatant, specifically dimers and decamers, was also increased after OLT1177 (Additional file 1: Fig. S2a–d). Moreover, after a short pulse (1 h) of stimulation with α-synuclein, OLT1177 reduced the amount of intact α-synuclein in the lysates by almost 50% (Fig. 2e), suggesting increased degradation. As autophagy is an important contributor to the degradation of α-synuclein [36], p62 and LC3B markers were evaluated. At 6 h post-stimulation with α-synuclein, we found no change in the degradation of p62 with or without OLT1177 (Fig. 2f). However, OLT1177 reduced the level of both LC3BII and the LC3BII/I ratio (Fig. 2g, h). To further evaluate the impact of OLT1177 on autophagy-controlled degradation of α-synuclein, we blocked the central autophagy protein ATG5 by siRNA. Here, we observed no change in the levels of α-synuclein in the lysates using OLT1177 (Fig. 2i), indicating that OLT1177, at least partly, increases the degradation of α-synuclein in microglia by improving its autophagic capacity.

### OLT1177 prevents the aggregation of α-synuclein in the brain

**Fig. 3:**
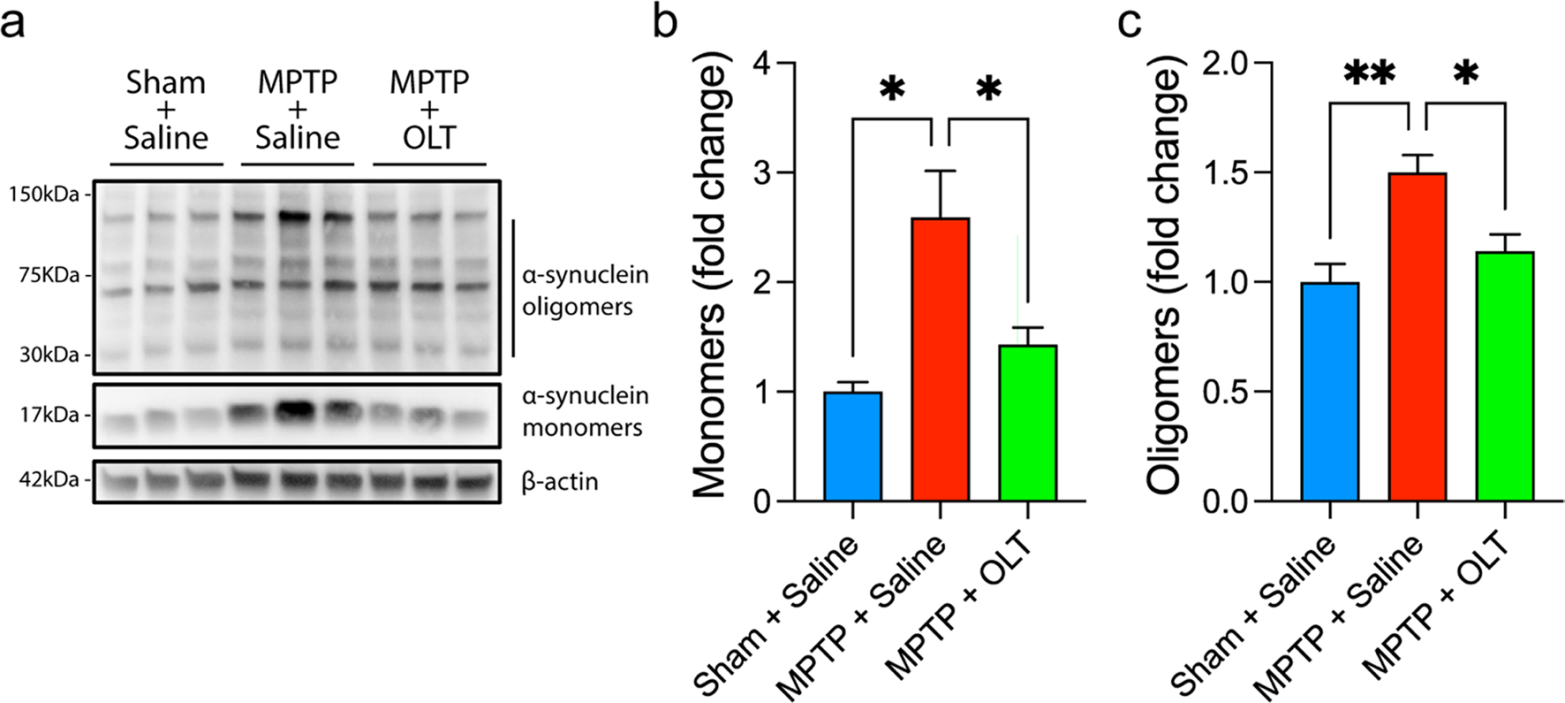
OLT1177 reduces the levels of α-synuclein in the ventral midbrain after MPTP-subacute administration. **a** Representative immunoblotting showing the levels of monomeric and oligomeric forms of α-synuclein in the different groups. Mice were treated with 200 mg/kg of OLT1177 (OLT) for 6 days. Saline was used as vehicle. β-actin was used as loading control. Molecular weight (kDa) is marked on the right side. **b** and **c** Quantification of monomeric (**b**) and oligomeric (**c)** forms of α-synuclein. *N* = 4 for Sham + Saline and *N* = 5 for MPTP + Saline and MPTP + OLT. One-way ANOVA with Tukey’s post hoc correction was used to analyze differences between groups. *p < 0.05 and **p < 0.01

Since MPTP promotes α-synuclein aggregation after a subacute regimen [37–39], we evaluated the effect of OLT1177 on the levels of this protein. 24 h after the last subacute injection of MPTP, mice were killed, and the levels of α-synuclein were determined by western blotting. VM was selected as the region to evaluate α-synuclein accumulation based on previous publications [40, 41]. MPTP induced a 2.5-fold increase in the monomeric form of α-synuclein (17 kDa) (Fig. 3a, b). This correlates with higher aggregation of various oligomeric forms of α-synuclein (150-30 kDa) (Fig. 3c). In agreement with our in vitro data, OLT1177 significantly reduced the levels of both monomers and oligomers of α-synuclein, maintaining levels close to basal (Fig. 3b, c).

### OLT1177 reduces the levels of pro-inflammatory cytokines in the brain

**Fig. 4:**
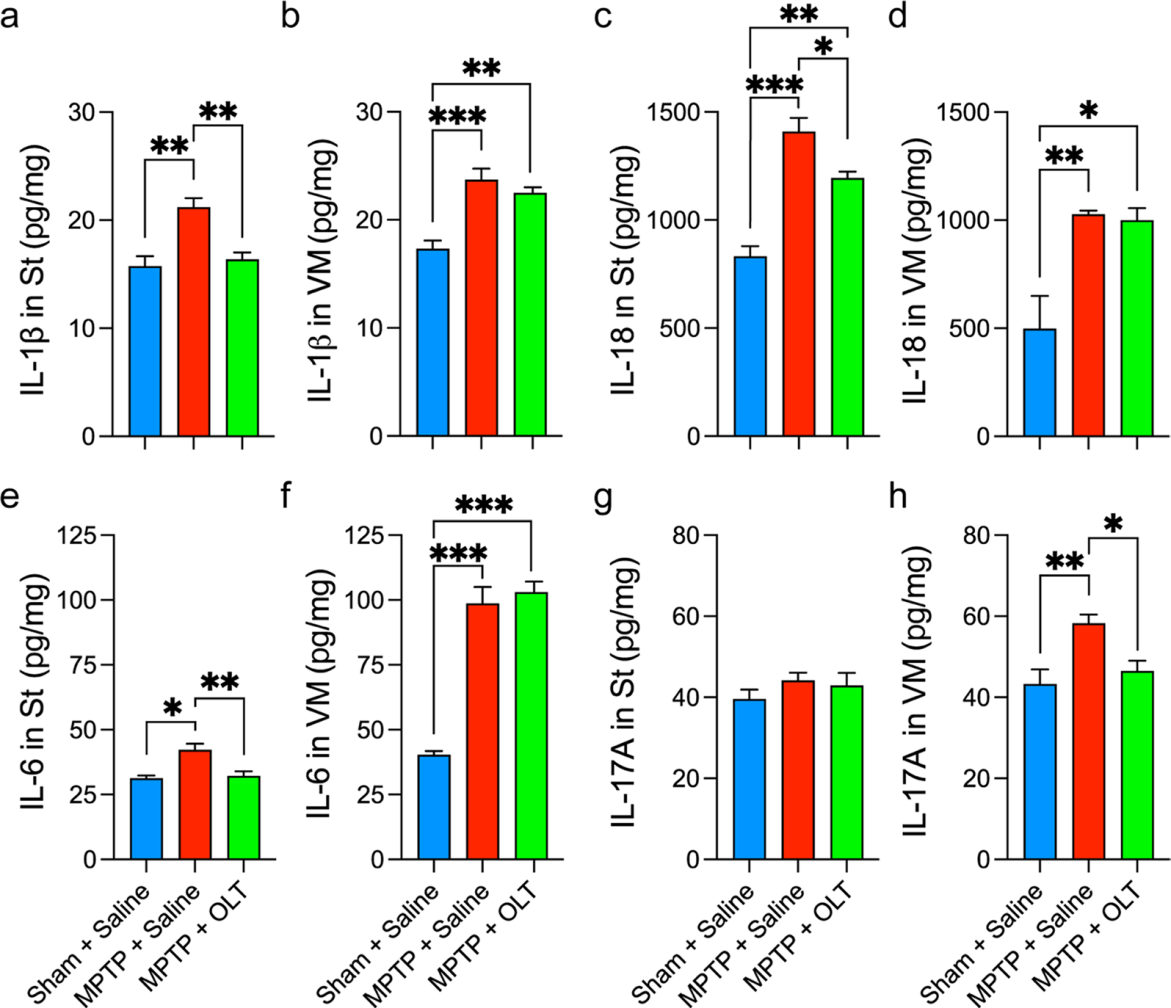
OLT1177 modulates the dynamics of pro-inflammatory cytokines in the brain after MPTP-acute administration. **a**–**d** Brain levels of IL-1β (**a** and **b**) and IL-18 (**c** and **d**) in the striatum (St) and ventral midbrain (VM) 4 days after MPTP administration. **e**–**h** Brain levels of IL-6 (**e** and **f**) and IL-17A (**g** and **h**) 7 days after MPTP administration. Mice were treated with 200 mg/kg of OLT1177 (OLT) once each day starting one hour before MPTP administration. Saline was used as vehicle. Data are represented as mean ± SEM. N = 4 per group for **a**–**d** and *N* = 5 per groups for **e**–**h**. One-way ANOVA with Tukey’s post hoc correction was used to analyze differences between groups. *p < 0.05, **p < 0.01, and ***p < 0.001

To evaluate the effect of OLT1177 at the cytokine level, we measured the levels of IL-1β and IL-18, which are the downstream cytokines of the NLRP3 inflammasome. IL-1β and IL-18 levels were measured in the St and VM at 4 days after MPTP-acute administration [42]. Levels of IL-1β significantly increased in the St and VM areas (Fig. 4a, b), confirming the activation of the NLRP3 inflammasome as proven in vitro. In the St, treatment with OLT1177 significantly reduced the levels of IL-1β by 24% (Fig. 4a), reaching basal conditions. In the VM, we detected no significant changes associated with OLT1177 (Fig. 4b). IL-18 levels were significantly elevated in both brain regions (Fig. 4c, d). OLT1177 induced a 15% reduction in the St (Fig. 4c), whereas no changes were observed in the VM (Fig. 4d).

After confirming that OLT1177 reduced IL-1β and IL-18, we measured IL-6 and IL-17A, downstream cytokines of IL-1β [43]. For IL-6, we observed that MPTP increased the levels in both areas of the brain after 7 days (Fig. 4e, f). OLT1177 significantly reduced the levels by 25%, reaching basal conditions in the St (Fig. 4e). No effects were detected in the VM (Fig. 4f). For IL-17A, we did not observe changes due to MPTP in the St (Fig. 4g); however, levels were significantly increased in the VM (Fig. 4h). This supports a previous report showing a similar dynamic of IL-17A in this area of the brain [44]. After treatment, OLT1177 significantly (*p* < 0.05) reduced the levels of IL-17A to nearly the same level as those in sham mice (Fig. 4h). Altogether, the data on cytokine dynamics demonstrated that MPTP induces an inflammatory response characteristic of activated NLRP3 and that OLT1177 effectively reduces the levels of these cytokines, modulating the inflammatory response towards baseline.

### OLT1177 protects dopaminergic neurons from MPTP-induced cell death

**Fig. 5:**
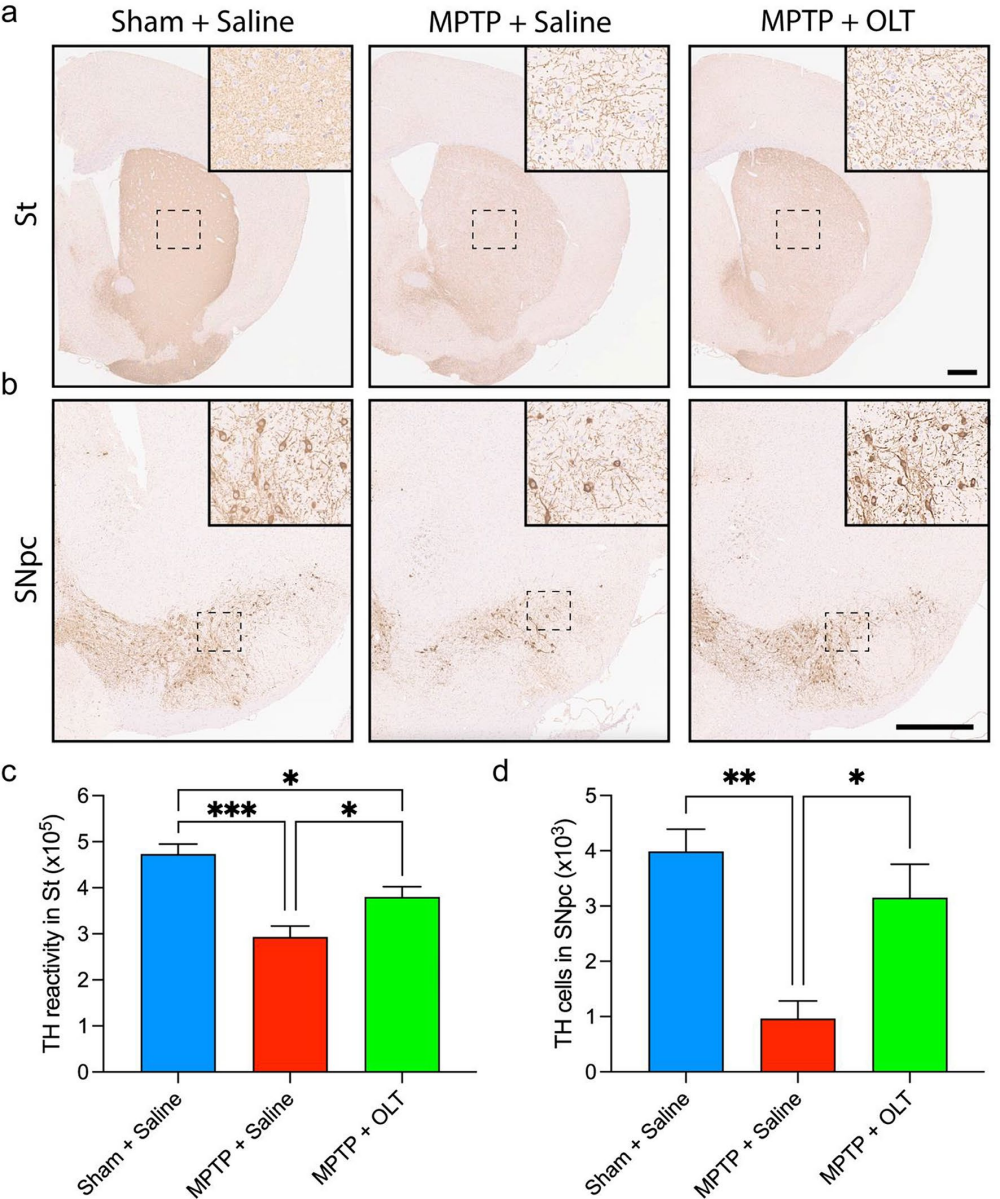
OLT1177 protects dopaminergic neurons from MPTP-mediated cell death. **a** and **b** Representative tyrosine hydroxylase (TH) immunostaining in the striatum (St, **a**) and substantia nigra pars compacta (SNpc, **b**). Mice were treated with 200 mg/kg of OLT1177 (OLT) for 7 days. Saline was used as vehicle. Positive signal appears in brown color. The area outlined in the box is shown in higher magnification in the inset. **c** and **d** TH immunoreactivity in the St (**c**) and TH stereological counting in the SNpc (**d**). Scale bar: 500 µm. Data are represented as mean ± SEM. *N* = 5 for Sham + Saline and *N* = 7 for both MPTP groups. One-way ANOVA with Tukey’s post hoc correction was used to analyze differences between groups. *p < 0.05, **p < 0.01, and ***p < 0.001

The effect of OLT1177 on the nigrostriatal pathway was evaluated by TH immunostaining 7 days after MPTP-acute administration, when the levels of dopaminergic neurons are lower [45]. As expected, administration of MPTP reduced the levels of St fibers by 45% compared to those in sham mice (Fig. 5a, c). In the SNpc, the number of positive neurons reduced from 4000 to 1000 (Fig. 5b, d). Treatment with OLT1177 protected dopaminergic neurons from the detrimental effects of MPTP. In the case of the St, mice treated with OLT1177 showed an increase (*p* < 0.05) in the density of TH-positive fibers compared with MPTP mice treated with saline (Fig. 5a, c). The number of TH-positive neurons in the SNpc also increased three-fold with OLT1177 treatment (Fig. 5b, d). In correlation with the reduction of α-synuclein shown above, these results demonstrate that OLT1177 protects dopaminergic neurons from MPTP-induced toxicity.

### OLT1177 reduced the astrogliosis and microgliosis reactivity associated to MPTP

**Fig. 6:**
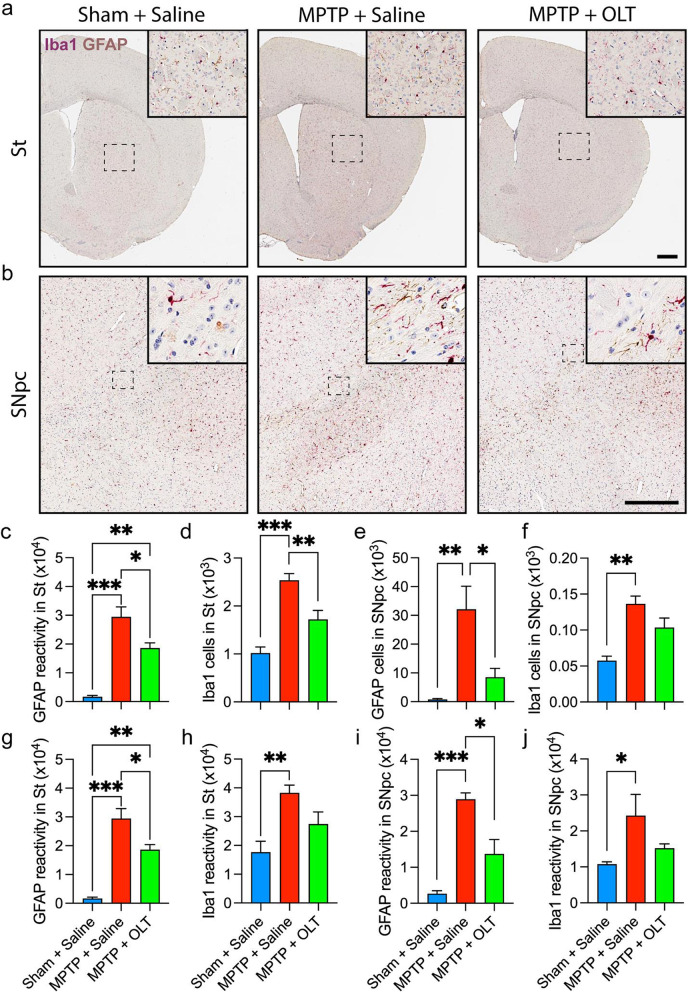
OLT1177 reduces astrogliosis and microgliosis reactivity induced by MPTP. **a** and **b** Representative glial fibrillary acidic protein (GFAP) and ionized calcium-binding adaptor molecule 1 (Iba1) immunostaining in the striatum (St, **a**) and substantia nigra pars compacta (SNpc, **b**). Mice were treated with 200 mg/kg of OLT1177 (OLT) for 7 days. Saline was used as the vehicle. Positive signal appears in purple for Iba1 and brown color for GFAP. The area outlined in the box is shown in higher magnification in the inset. **c**–**f** GFAP and Iba1 stereological counting in the St (**c** and **d**) and in the SNpc (**e** and **f**). **g**–**j** GFAP and Iba1 immunoreactivity in the St (**g** and **h**) and in the SNpc (i and **j**). Scale bar: 500 µm. Data are represented as mean ± SEM. *N* = 5 for Sham + Saline and *N* = 7 for both MPTP groups. One-way ANOVA with Tukey’s post hoc correction was used to analyze differences between groups. *p < 0.05, **p < 0.01, and ***p < 0.001

After evaluating the changes in the levels of dopaminergic neurons, we characterized the microglial and astrocytic reactivity in the same areas. In the St, we found that OLT1177 significantly reduced the amount of GFAP-positive cells and area in comparison with MPTP mice treated with saline (Fig. 6a, c, g). We also observed a reduction in the number of Iba1-positive cells with a tendency to decrease immunoreactivity (Fig. 6a, d, h). In the case of the SNpc, we observed a similar pattern, with OLT1177 significantly decreasing the levels of GFAP (Fig. 6b, e, i) and slightly reducing the number and area of Iba1-positive cells (Fig. 6b, f, j). Collectively, these data support the cytokine data from the same areas, showing a reduction in the MPTP-associated inflammatory response by OLT1177.

### OLT1177 preserves the levels of TREM2 in the striatum

Recent findings propose myeloid cell-triggered receptor II (TREM2) as a potential target in the treatment of PD [46], thus, we measured TREM2 levels after OLT1177 treatment. Protein levels of TREM2 were measured in the St area of the brain on day 4 after MPTP-acute administration. MPTP exposure resulted in a 25% reduction in the levels of the non-glycosylated form of TREM2 (28 kDa) (Additional file 1: Fig. S3a, b), whereas treatment with OLT1177 normalized these levels (Additional file 1: Fig. S3a, b). Investigating the 35-kDa glycosylated form of TREM2, we found no significant changes (Additional file 1: Fig. S3a, c). Although a tendency towards elevated 35-kDa glycosylated TREM2 levels was observed after MPTP, the differences were not significant.

## Discussion

Parkinson’s disease is the second-most common neurodegenerative disorder after AD; however, few successful interventions have surfaced since the use of levodopa was approved 60 years ago [47]. Newer advances are mostly aimed at reducing the peripheral metabolism of levodopa and avoiding the processing of the natural DA in the brain [48, 49]. Excluding those, the remaining current clinical trials include DA receptor agonists, convalescent plasma therapy, cell-based treatments, gene-based therapies, serotonin compensation, anti-apoptotic drugs, and therapies against α-synuclein aggregation [50, 51]. Although there is increasing evidence that neuroinflammation contributes to the neurodegenerative features of PD [52], there are no clinical trials specifically aimed towards reducing inflammation in PD. Based on the data above, we propose NLRP3 inhibition using OLT1177 as a novel therapeutic agent to diminish the pathological features of PD.

OLT1177 is a selective inhibitor of the NLRP3 inflammasome. As it has been previously described, the NLRP3 inflammasome is linked to microglia-mediated inflammation in PD [52, 53]. For example, in post-mortem brains from PD patients, *NLRP3* gene expression is elevated in dopaminergic areas and correlates with reduced TH levels [54]. Furthermore, loss-of-function polymorphisms in the *NLRP3* gene are associated with a decreased risk of PD [54].

The relationship between NLRP3 activity and PD can be explained by the release of α-synuclein from the dying dopaminergic cells. The pathological aggregates of α-synuclein provides the first “priming” signal for the activation of NLRP3 through the TLR2/NF-κB pathways, leading to the transcription of *NLRP3*, *IL1b*, and *IL18* genes [55, 56]. In addition to “priming” microglia, aggregated α-synuclein also impairs mitochondrial function following microglial engulfment [57]. This impairment leads to a breakdown and release of mitochondrial DNA as well as the generation of ROS, each of which provides the second “activation” signal for the assembly of the NLRP3 inflammasome. Activated NLRP3 results in the subsequent caspase-1-mediated release of mature IL-1β and IL-18 [21, 58].

This activation of NLRP3 by α-synuclein plays a role not only in the induction of an inflammatory response, but also in the further aggregation and propagation of α-synuclein. For example, activated microglia have been associated with impaired autophagy, which in turn contributes to α-synuclein aggregation [59]. Supporting this, we found that the OLT1177-induced clearance of recombinant α-synuclein was dependent on the autophagy pathway, suggesting that NLRP3 inhibition, and thus anti-inflammatory interventions, could restore the autophagy clearance capacity. A detailed study on the transmission of α-synuclein via microglial exosomes demonstrated an increase in α-synuclein aggregation in neurons when combined with pro-inflammatory cytokines [60]. Therefore, inflammatory cytokines have the potential to exacerbate α-synuclein aggregation and induce dopaminergic cytotoxicity, contributing to PD [61]. Given these data, blockade of the NLRP3 inflammasome is a promising approach for the treatment of PD, and includes OLT1177, as discussed below.

In the case of PD, targeting the NLRP3 inflammasome in mouse models has been addressed using various approaches. For example, NLRP3 deficiency protects against nigrostriatal degeneration of dopaminergic neurons in an MPTP model of PD [22, 23]. In addition, NLRP3 deficiency also improved the autophagy of α-synuclein, reducing its transmission to other neurons [23]. Pharmacological inhibition of NLRP3 resulted in improved features in a progressive 6-OHDA model of PD [62]. Each approach correlated with reduced inflammatory response in brain areas, demonstrating the critical role of NLRP3 in the inflammatory response of PD [22, 23, 62].

In the present study, we used the neurotoxic MPTP model to study the role of OLT1177 in PD. We observed that daily administration of 200 mg/kg of OLT1177 reduced the levels of α-synuclein and inflammatory markers in the brain, which correlated with the increased survival of dopaminergic neurons in the nigrostriatal pathway. This was confirmed by an increase in microglial clearance capacity after OLT1177 exposure in vitro. Microglia and astrocytes reactivity was also reduced after treatment with OLT1177. Changes in astrocytes are explained by an indirect effect of microglia [63] since the expression and functionality of the NLRP3 inflammasome are restricted to microglia, but not astrocytes, in the mouse brain after stimulation [64]. At the motor level, we demonstrated that OLT1177 prevented the functional deficits associated with MPTP neurotoxicity using the rotarod device. We also observed that OLT1177 protected against the effects of MPTP in peripheral blood by attenuating systemic inflammation, which has been described to contribute to dopaminergic cells loss [65].

In addition to reducing the levels of IL-1β and IL-18 in the brain, OLT1177 reduced the levels of IL-6 and IL-17A. IL-17A has recently been associated with detrimental effects in a mouse model of PD [44, 66]. IL-17A deficiency or blockade prevents dopaminergic neurodegeneration and motor impairments in MPTP models [44]. The opposite effects were observed with the adoptive transfer of effector T cells, demonstrating the contribution of the adaptive immune response to the pathogenesis of PD [44].

Similar to IL-17A, TREM2 has recently been proposed to contribute to PD [46]. Numerous findings report that loss-of-function mutations in TREM2 are associated with increased risk of PD [67, 68]. TREM2 is highly expressed on the surface of microglia and participates in survival, proliferation, phagocytosis, and expression of inflammatory factors [69]. In different models of PD, TREM2 deficiency aggravated neuroinflammation and neurodegeneration [70], whereas its overexpression protected dopaminergic neurons from MPTP toxicity [71]. Our data showed that NLRP3 inhibition by OLT1177 increased the levels of TREM2 in the St of MPTP mice, correlating with the preservation of dopaminergic neurons. These changes in TREM2 expression did not affect its glycosylation pattern, which also affects its performance, as observed in AD [72].

Although there are reports in models of PD using mice deficient in the *Nlrp3* gene [22, 23] as well as other NLRP3 inhibitors [62], what distinguishes our findings is the use of an effective drug that is safe in humans [24–26]. OLT1177 has been reported to be safe and effective in Phase Ib clinical trials for heart failure [26] and Phase IIa for gout flares [25]. Moreover, oral efficacy of OLT1177 has been shown in mouse models of arthritis [73], multiple systemic sclerosis [27], Alzheimer’s disease [29], and melanoma [74].

## Conclusions

In conclusion, the data in our study support the critical role of NLRP3 in the pathological progression of PD. We found that OLT1177 is safe in mouse models of PD, crosses the BBB, and effectively minimizes the features of PD, including reduction in α-synuclein aggregates and pro-inflammatory markers. All these changes translated into the protection of dopaminergic neurons and the improvement of locomotor performance. These results provide a rationale for future investigations on NLRP3 inhibition by OLT1177 in PD.

## Supplementary Information

**Additional file 1: Figure S1.** OLT1177 crosses the blood–brain barrier and reaches therapeutic concentrations. **Figure S2.** OLT1177 increases the clearance of α-synuclein oligomers by microglia in vitro. **Figure S3.** OLT1177 increases the levels of TREM2 after MPTP-acute administration.

## Data Availability

The datasets used and/or analyzed during the current study are available from the corresponding author on reasonable request.

## Abbreviations

AD: Alzheimer’s disease
BBB: Blood–brain barrier
DA: Dopamine
GFAP: Glial fibrillary acidic protein
Iba1: Ionized calcium-binding adaptor molecule 1
MPTP: 1-Methyl-4-phenyl-1,2,3,6-tetrahydropyridine
NLRP3: NOD-like receptor pyrin domain containing protein 3
PD: Parkinson’s disease
SNpc: Substantia nigra pars compacta
St: Striatum
TH: Tyrosine hydroxylase
TREM2: Myeloid cell-triggered receptor II
VM: Ventral midbrain
