# Supplementary material for: Pharmacologic inhibition of NLRP3 reduces the levels of α-synuclein and protects dopaminergic neurons in a model of Parkinson’s disease
Source: J Neuroinflammation. 2023 Jun 22;20:147. doi: 10.1186/s12974-023-02830-w (PMC10286423; doi:10.1186/s12974-023-02830-w)
Supplement: Supplementary file 1 — Additional file 1: Figure S1. OLT1177 crosses the blood–brain barrier and reaches therapeutic concentrations. Figure S2. OLT1177 increases the clearance of α-synuclein oligomers by microglia in vitro. Figure S3. OLT1177 increases the levels of TREM2 after MPTP-acute administration. [file 12974_2023_2830_MOESM1_ESM.docx]

Additional file 1


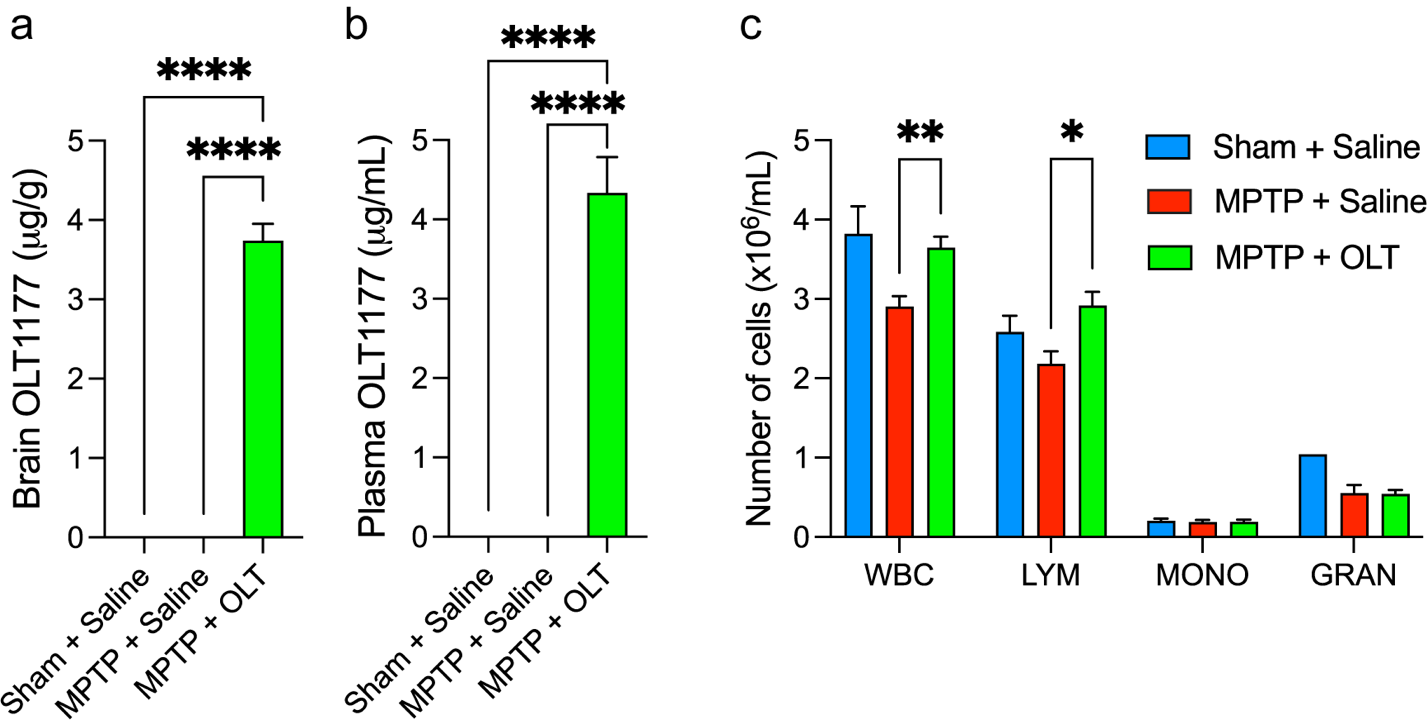


Additional file 1: **Figure S1:** OLT1177 crosses the blood-brain barrier and reaches therapeutic concentrations. (a-b) Brain (a) and plasma (b) levels of OLT1177 at day 7 after MPTP-acute administration. Mice were treated daily with 200 mg/kg of OLT1177 (OLT). Samples were collected 24 hours after the last OLT1177 injection. (c) Number of different cell types in the blood after treatment with 200 mg/kg of OLT1177 (OLT) in MPTP-injected mice. White blood cells (WBC), lymphocytes (LYM), monocytes (MONO), and granulocytes (GRAN) were measured from the peripheral blood at day seven after MPTP administration. Data are represented as mean ± SEM. N = 5 per group in (a). N = 5 for Sham + Saline and N = 6 for MPTP + Saline and MPTP + OLT in (b). One-way ANOVA for (a, b) and two-way ANOVA with Tukey’s post hoc correction for (c) were used to analyze differences between groups. * p < 0.05, ** p < 0.01, and *** p < 0.001.


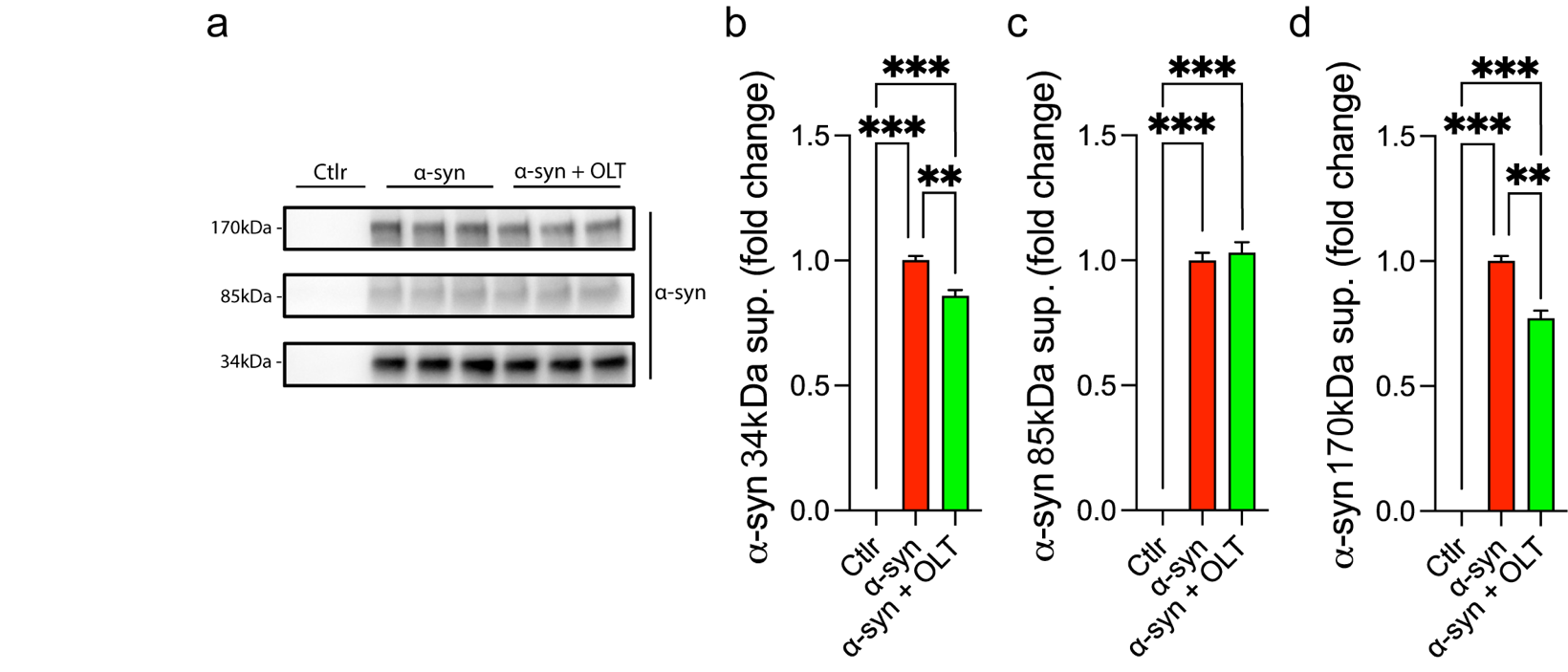


Additional file 1: **Figure S2:** OLT1177 increases the clearance of α-synuclein oligomers by microglia *in vitro*. (a) Representative immunoblotting showing the levels of α-synuclein dimers (34kDa), pentamers (85kDa), and decamers (170kDa) in the supernatant of neonatal microglia in culture. Cells were treated with OLT1177 (OLT, 10µM) one hour before stimulation with recombinant α-synuclein (α-syn, 10µM). Molecular weight (kDa) is marked on the right side. (b-d) Quantification of the levels of dimers (b), pentamers (c), and decamers (d) at 6h. Data are pooled from 3 separate experiments and are represented as the mean ± SEM. One-way ANOVA with Tukey’s post hoc correction was used to analyze differences between groups with * p < 0.05, ** p < 0.01, and *** p < 0.001.


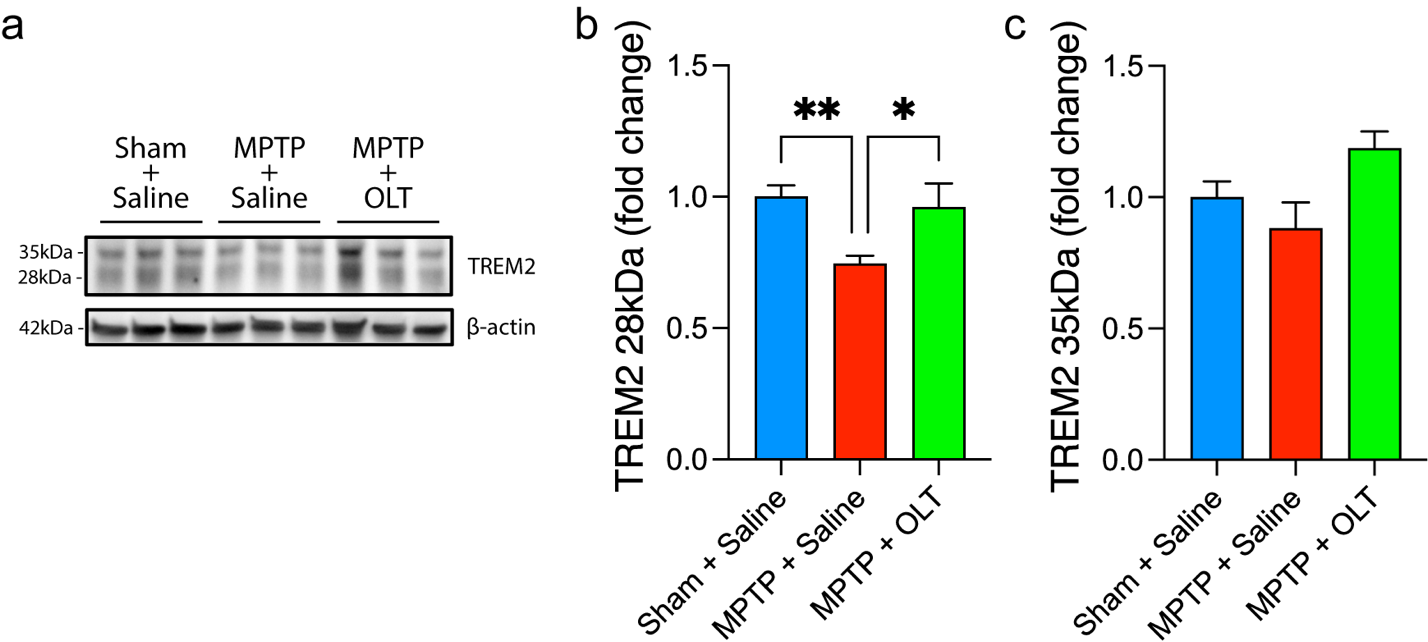


Additional file 1: **Figure S3:** OLT1177 increases the levels of TREM2 after MPTP-acute administration. (a) Representative immunoblotting showing the levels of glycosylated (35kDa) and non-glycosylated (28kDa) forms of TREM2 in the striatum 4 days after MPTP administration. Mice were treated with 200 mg/kg of OLT1177 (OLT) once each day starting one hour before MPTP administration. β-actin was used as loading control. Molecular weight (kDa) is marked on the right side. (b and c) Quantification of 28kDa (b) and 35kDa (c) forms of TREM2. N = 4 per group. One-way ANOVA with Tukey’s post hoc correction was used to analyze differences between groups. * p < 0.05 and ** p < 0.01.
